# A Brief Spanish Version of the Child and Adolescent Mindfulness Measure (CAMM). A Dispositional Mindfulness Measure

**DOI:** 10.3390/ijerph16081355

**Published:** 2019-04-15

**Authors:** Joan Guerra, María García-Gómez, Jorge Turanzas, Jose R. Cordón, Cristina Suárez-Jurado, José Miguel Mestre

**Affiliations:** 1 Departamento de Psicología, Universidad de Extremadura, 10071 Cáceres, Spain; joangb@unex.es; 2 Departamento de Psicobiología, Universidad de Murcia, 30001 Murcia, Spain; mgg99158@um.es; 3 Universidad de Educación a Distancia (UNED), Centro de Algeciras, 11202 Cádiz, Spain; jturanzas@algeciras.uned.es; 4 Instituto de Investigación y Desarrollo Social Sostenible (INDESS), Universidad de Cádiz, Jerez de la Frontera, 11405 Cádiz, Spain; josercordon@gmail.com; 5 ELEA, Centro de Psicología y Logopedia, Campo de Gibraltar, La Línea de la Concepción, 11300 Cádiz, Spain; crsuju@hotmail.com

**Keywords:** dispositional mindfulness, mindfulness measurement in children, CAMM, children and adolescents

## Abstract

Dispositional Mindfulness (DM) is the awareness of the thoughts and feelings in the present moment. DM in children and adolescents has been related to mechanisms of change in mindfulness-based interventions, which have shown significant mediation relationships with mental health outcomes (for instance, lower social anxiety, depression symptoms, or perceived stress). However, the assessment of DM among children and adolescents is being unsatisfactory due cultural biases and/or reliability issues. In this study, we examined the psychometric properties of the Spanish version of the Child and Adolescent Mindfulness Measure (CAMM) in a sample of 687 children and adolescents between 8 and 16 years old. Although the CAMM has been validated in English, Portuguese, Italian, and Catalonian versions, until now no data has been reported in a Spanish context. Results showed that the best CAMM factor structure was constituted by five items from the original version (1, 4, 7, 8, and 9). These items defined dispositional mindfulness. The rest of the items (2, 3, 5, 6, and 10) were eliminated from the Spanish final version. The analyses revealed good reliability and internal consistency for the Spanish version of the CAMM. As we expected, the confirmatory factor analysis showed the unidimensional structure of the CAMM.

## 1. Introduction

Mindfulness is defined as a process of bringing a certain quality of attention to moment-by-moment experience that starts with bringing awareness to current experience by regulating the focus of attention [1]. However, dispositional mindfulness (DM) has been defined as awareness of the thoughts and feelings in the present moment [2]. Therefore, DM is a predisposition or trait for living in a mindfulness way [3]. 

A systematic review conducted by Tomlinson et al. pointed out how DM was related to the psychological health of young people. Authors reviewed non-interventional and quantitative DM’s articles in non-clinical samples [4]. According to their revision, DM was negatively related to non-adaptative emotions (such as anxiety or depression symptoms), and positively linked to adaptative cognitive strategies of emotional regulation processes (such as reappraisal and acceptance) and positive emotions (f. i., happiness) [5]. With adolescents’ samples, DM was positively related to subjective well-being [6], but mostly DM showed negative relationships with lower levels of dysphoric mood and better tolerance to the effects of stress [7], lower social anxiety [8], even in gifted adolescents, higher levels of DM corresponds to lower levels of depression, anxiety, and negative emotions [9].

Nowadays, innovations in psychological treatment have seen an increase in the use of mindfulness intervention approaches [9]. Several mindfulness-based programs have become increasingly popular, such as mindfulness-based stress reduction (MBSR) [10,11]; mindfulness-based cognitive therapy (MBCT) [12,13,14]; and acceptance and commitment therapy (ACT) [15]. 

In the academic literature, the psychological use of mindfulness based on interventions (MBI) has been mainly focused on adult populations [16]. MBI studies have shown efficacious approaches to promoting psychological health and well-being [17]. When applying MBI to adolescents and children, two recent meta-analysis [4,18] reported that mindful trainings led to positive effects on their psychological functioning (see also [19,20]), propitiating reduction of some key psychological problems such as depression, and anxiety, facilitating externalization of problems, improved attention, and better academic achievement and MBI also increased the efficacy of psychological disease treatments. MBI and activities for children and adolescents have a potential mediating role for increasing adolescents’ emotional regulation and well-being [14,18,21,22,23], making that person less likely to be caught up with thoughts about the past (rumination) or future (worry) [23].

However, among children and adolescents these findings should be considered tentative due to the fact some measures of DM at this age display both cultural biases and reliability issues, for instance the Spanish version of the Children and Adolescent Mindfulness Measure (CAMM) [24]. Although there are different approaches to the assessment of DM in children and adolescents, most DM measures have suggested that mindfulness is a unitary construct [25] with two important dimensions: awareness (or presence) [26] and acceptance (or non-judging) [27].

There are some Spanish measures of DM developed for adolescent and children (see for example [28,29]). Nonetheless, we chose CAMM [30] for being both shorter than others and the fact it has worldwide acceptance among authors [27]. Greco et al. created the first mindfulness questionnaire developed for children and adolescents, the Child and Adolescent Mindfulness Measure (CAMM), a 25-item version, with three-factors: observing, paying attention to the sensations of the body, and the avoidance of emotions. Their last version conducted a confirmatory factor analysis found a single-factor solution with 10 items [30]. 

Some non-English validations of the CAMM found similar psychometric properties as in Greco et al.’s last version [30]. For instance, the CAMM validation on Dutch children and adolescent samples (*n*_1_ = 275, 10–12 years and *n*_2_ = 560, 13–16 years) showed a single factor with good fit. However, the authors also found a similar CAMM first component for children and adolescents (“present-moment non-judgmental awareness”), but the second component for children (“suppressing or avoiding thoughts and feelings”) and adolescents (“distractibility or difficulty paying attention”) was named differently according to the age of sample. Therefore, the age could change the meaning of the DM. Besides, the ten-item CAMM reliability increased among adolescents (α = 0.71 for children, and α = 0.80 for adolescents). Authors reported comparable psychometric properties with the original CAMM and two factors: “mindful awareness” and “being non-judgmental” [31]. Two independent studies were conducted to validate and assess the psychometric properties of the French-Canadian version of the CAMM [32], with French and Indigenous youth. Authors reported an exploratory and a confirmatory factor analysis validated a single factor. Their findings revealed no variance issues due to both floor and ceiling effect, which means that French-Canadian CAMM version was not easy or difficult to answer. 

However, CAMM studies with similar (Latin-European) cultural samples to Spanish ones reported better psychometric properties of the CAMM when it was considered a single factor instead of two. For example, both the Portuguese [33] and the Catalan validation of the CAMM [34] performed an exploratory and confirmatory factor analyses and found a 10 item solution with one factor. However, using Item Response Theory, the Italian version of the CAMM [35] suggested that Greco et al.’s original version of the CAMM does not fit due to unsatisfactory psychometric properties of two items. When both items are removed, the eight-item CAMM version provided better reliability and the confirmatory factor analysis consolidated the one-dimensional structure, with significant positive correlations with emotional intelligence and quality of life, and significant negative correlations with symptoms of somatizing disorder. This Italian version matched a previous Spanish validation Therefore, it is possible to find a DM tool with a unique factor and stronger psychometric properties reducing the number of items and validating its predictive validity.

Our aim was to explore whether a shorter version the Spanish version of the Child and Adolescent Mindfulness Measure (CAMM) provides both a better internal consistency and predictive validation with health-psychological outcomes. We expected DM scores of the CAMM (positively recoded) will correlate positively with positive affect and negatively with negative affect and thought suppression. We will also provide compared predictive results among ten, eight, and final-item versions of the CAMM.

## 2. Materials and Methods 

### 2.1. Participants

We randomly selected 687 children and adolescents from elementary schools of two regions in Spain (Valencia *n*_1_ = 360, *M*_age_ = 12.68, *SD*_age_ = 1.52, 51% of female participants and Andalusia *n*_2_ = 318, *M*_age_ = 11.25, *SD*_age_ = 2.20, 49.1% of female participants). Participants who voluntary agreed to be included had to bring a parent-signed consent (according to Spanish Organic Law of Data Protection) and later they received a formal information about their personal results. Written informed consent was obtained from the parents/legal guardians of all participants. According to Research and Ethical Commission of the Institute for University Research on Social and Sustainable Development (INDESS, University of Cadiz, Spain), we had to follow certain ethical recommendations: (a) all participants had to bring an informed consent from their parents, especially minors under 14 years old; (b) we had to inform and receive permission from every single parent’s student school association, and (c) the study had to be approved by an external ethical board (in this case the Ethical Board of the University of Cadiz, Spain). For this reason, the study was compliant with the following ethical standards: the 1964-Helsinki Declaration and its later amendments or comparable ethical standards and, according to the Article 13.1 of the Spanish Organic Law of Data Protection, the “data of persons over fourteen years of age may be processed with their consent, except in those cases in which the Law requires the assistance of the holders of parental authority or guardianship. In the case of minors under 14 years of age, the consent of the parents or guardians will be required”.

### 2.2. Measures

#### Dispositional Mindfulness

To assess mindfulness, we used the Child and Adolescent Mindfulness Measure (CAMM; [30]). The original CAMM consist of 10 items, responded to on a 5-point Likert scale, ranging from 0 (never true) to 4 (always true). This instrument has reported that the mean score of CAMM was 22.73 (*SD* = 7.33) with a Cronbach’s alpha (α) of 0.81 [30]. Lower scores would indicate a disposition for having mindful skills in everyday life. This measure is based on the Kentucky Inventory of Mindfulness Skills (KIMS) [36], which assesses acting with awareness of the present moment and accepting without judgment. Due to items were written in a negative sense, we also reverted the scoring for an easier interpretation. Hence, higher scores indicated higher DM. 

### 2.3. Criteria

#### 2.3.1. Thought Suppression and Intrusion Using the White Bear Suppression Inventory (WBSI)

Based in previous ideas about the effect of thought suppression on mental health [37,38], this instrument comprises 15 items to evaluate chronic thought suppression tendencies [37]. The internal consistency measured with Cronbach’s alpha was 0.86. The respondents are requested to indicate their agreement with statements on a 5-point Likert scale ranging from 1 ‘strongly disagree’ to 5 ‘strongly agree’. Thus, the total score ranges from 15 to 75, with higher scores indicating greater tendency to suppress undesirable thoughts. It contains statements such as “There are things I prefer not to think about” or “I always try to put problems out of mind”. The WBSI has demonstrated high internal consistency in Spanish (and Portuguese) samples [39]. This inventory is an indicator of the frequency individuals have intrusive and ruminative thoughts, and has been found to correlate positively with depressive symptoms, anxiety, and obsessive-compulsive behavior [40]. For this study we used average scores (from 1 to 5). According the last recommendations, two of WBSI’s factors were used (six items in each one), “Suppression” and “Intrusion” thoughts [37]. 

#### 2.3.2. The Positive and Negative Affect Schedule for Children (Spanish Validation ‘PANASN’)

The Spanish PANASN [41] was based on the original instrument [42]. This is a 30-item measure for children and young adolescents, which assesses Positive affects (PA; e.g., cheerful) and Negative affects (NA; e.g., lonely) using 15 items each. PANASN also provides a measure of *Balance* (PA–NA). The psychometric properties indicated good reliability with a Cronbach’s alpha of 0.88 for PA and 0.87 for NA. Participants were asked to describe how they felt during the past few weeks on a 5-point Likert scale ranging from 1 ‘slightly or seldom’ to 3 ‘much or often’. Spanish PANAS-C (PANASN) has shown appropriate values of internal consistency, as well as convergent and discriminant validity [41,42]. For this empirical study, an average PANASN score was used (from 1 to 3). 

### 2.4. Procedure

All the children were recruited in ACES. After an introductory session with the parents, a total of 22 families agreed to participate in our study. The parents received a calendar with the sessions of the APAC program (see below) and the content of the sessions, as well as an agreement detailing the conditions for their children’s participation in the study. All data concerning the participants were treated in accordance with these conditions and with the full consent of the parents. All parents received an individualized report from their child for each evaluation conducted in the study.

### 2.5. Statistical Analysis

To determine the best way to analyze and improve the psychometric characteristics of CAMM, an Exploratory Factor Analysis was performed with the Valencia sample (*n*_1_ = 360), and the correspondent Confirmatory Factor Analysis was run with the Andalusia sample (*n*_2_ = 318). To avoid validity problems, and redundant results, several Exploratory Factor Analysis (EFA) were performed on the Valencian sample. Two models emerged, using the 10-item CAMM scale (see Appendix A), with three possible factors, and another with only one factor. We used the Andalusian sample for performing a valid Confirmatory Factor Analysis (CFA) using IBM AMOS software (Version 23) (Armonk, NY, USA) to evaluate the goodness-of-fit on this sub-sample. No problems about normality, missing values or outliers were detected in either samples. Then, a Confirmatory Factor Analysis was performed on the Andalusia sample, to assess the goodness-of-fit of the model that come out from the Valencia sample.

## 3. Results

Our primary goal was to determine if the CAMM may be reduced to a shorter measure with better psychometric properties and less cultural biases. 

### 3.1. Exploratory Factor Analysis of the Spanish 10-Items CAMM

To determine whether original CAMM could be reduced, other EFAs were performed. A first solution showed a 7-item CAMM (items # 2, 5, and 10 were dropped due to negative item-scale correlation) in the Valencian sample. A principal component analysis (PCA) was conducted on the seven items with oblique rotation (promax), this rotation method allows factors to be correlated. The Kaiser–Meyer–Olkin measure verified the sampling adequacy for the analysis, KMO = 0.781 is considered good [43,44,45], and all KMO values for individual items were >0.55, which is above the acceptable limit of 0.5 [43]. Bartlett’s test of sphericity χ² (21) = 819.5, *p* < 0.001, indicated that correlations between items were sufficiently large for PCA. An initial analysis was run to obtain eigenvalues for each component in the data. Two components had eigenvalues over Kaiser’s criterion of 1 and in combination explained 63.9% of the variance. Table 1 presents the initial extraction and variance explained.

**Figure 1:**
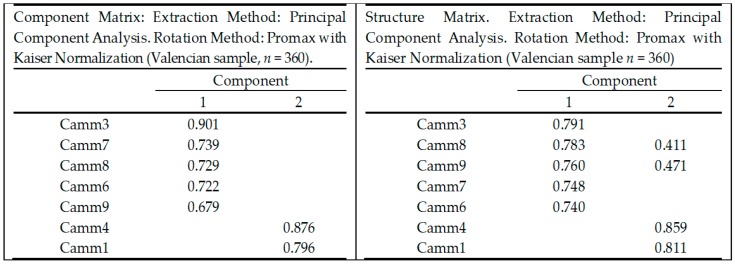
Component matrix and structure after exploratory factor analysis (EAF) using Valencian sample (*n*_1_ = 360). After EFA, seven items finally remained.

To decide whether we should retain one or two factors, a graphic analysis was used. The scree plot was slightly ambiguous and showed inflexions that would justify retaining both components. Given the large Valencian sample size (*n*_1_ = 360), and the convergence of the scree plot and Kaiser’s criterion on two components, this is the number of components that were retained in the final EFA analysis. Figure 1 shows both the pattern and the structure matrices of this EFA. To assess invariance across samples, a separate EFA was performed on Andalusian sample, showing only one component and explaining 44.6% of total variance, based on Kaiser’s criterion. Component and structure matrix were similar to those on Valencia sample, but with one component only. This finding is consistent with the CFA performed in this sample using SEM.

### 3.2. Confirmatory Factor Analysis

**Figure 2:**
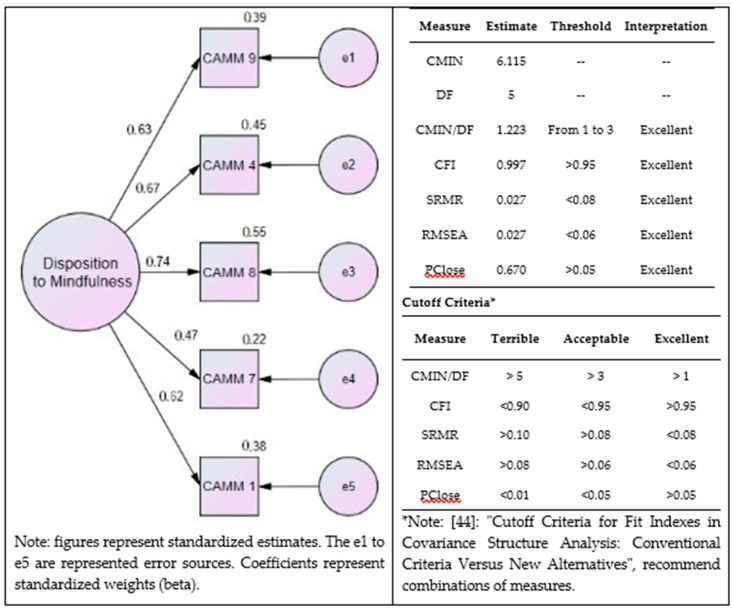
Confirmatory factor analysis (CFA) in Andalusian sample. Final version proposed as Spanish 5-items CAMM.

To confirm the factor structure carried out on the Valencian sample, several CFA representations were tried in AMOS, but no single one could fit a valid one in the Andalusian sample, so finally a one-component model came up with only five items that fits in an excellent way our data. Figure 2 summarizes this model, and states the goodness-of-fit of the model. Items removed were by loading criteria, until an available model was reached, so two other items have to be removed from the final model. The final model comprises items #1, 4, 7, 8 and 9.

Due to the sample size (*n*_2_ = 318), also we looked at CMIN/DF= 1.223 (chi square/degree of freedom ratio). Different researchers [46,47] have recommended using a ratio as low as 2 or as high as 5 to indicate a reasonable fit. A comparative fix index (CFI; in our case 0.997) close to 1 indicates a very good fit, > 0.9 or close to 0.95 indicates good fit, by convention, CFI should be equal to or greater than 0.90 to accept the model, and CFI is independent of sample size. Root mean square error of approximation (RMSEA = 0.027); the RMSEA values are classified into four categories: close fit (0.00–0.05), fair fit (0.05–0.08), mediocre fit (0.08–0.10), and poor fit (over 0.10). PCLOSE = 0.670 tests the null hypothesis that RMSEA is no greater than 0.05. If PCLOSE is less than 0.05, we reject the null hypothesis and conclude that the computed RMSEA is greater than 0.05, indicating lack of a close fit. Standardized root mean square residual (SRMR) is an absolute measure of fit and is defined as the standardized difference between the observed correlation and the predicted correlation. It is a positively biased measure and that bias is greater for small N and for low degrees of freedom studies.

### 3.3. Reliabilities and Construct Validity

This new reduced 5-item scale had an improved reliability of Cronbach’s alpha α = 0.763 using the whole sample. Regarding construct validity, this model presents Convergent Reliability, CR0 = 0.765, well above the recommended threshold of 0.5 [47], the Average Variance Extracted (AVE0 = 0.4) is slightly under 0.5. However, Malhotra and Dash argued that AVE is often too strict, and reliability can be established through CR alone [46]. The Maximum Reliability, MaxR(H), presents a very good value of 0.78 [43], therefore the 5-item Spanish CAMM has a good construct validity. 

To verify the sampling adequacy (*n* = 678), the 5-item Spanish CAMM showed a good KMO measure (KMO = 0.818). Besides, all KMO values for individual items were above 0.6, where the acceptable limit is 0.5 [43]. Bartlett’s test of sphericity χ² (10) = 749.207, *p* < 0.001, indicated that correlations between items were sufficiently large for PCA. The PCA showed one component with eigenvalues over Kaiser’s criterion of 1 and in combination explained 51.69% of the variance. Next, the relevant statistics are presented for the whole sample.

### 3.4. Predictive Validity Comparison among CAMM Versions

Cronbach’s alpha for the 10-item scale was α = 0.67, however, items 2, 5 and 10 showed very low item-scale correlations (0.17; 0.18; −0.37; respectively), excluding those items, the new Cronbach’s alpha was α = 0.77, all items now presenting item-scale correlations just or well above 0.40. Although, this 7-item CAMM version was only used during the EFA processes because it was not reasonable to include next CAMM items—# 2, 5 and 10. Table 2 shows descriptive and correlations among variables and CAMM.

### 3.5. Predictive Regression Analysis

We conducted two linear regressions between the reduced 5-item CAMM version (DM measure) and both Negative Affect (NA) of PANASN and Thought Suppression (TS) of WBSI. Regarding TS, DM predicted 37.2% (R^2^) of the explained variance with WBSI total score (F(1, 316) = 187.44, β = −0.61, (t = −13.69, *p* < 0.001)). Regarding NA, DM predicted 21% (R^2^) of the explained variance with NA score of PANASN (F(1, 316) = 83.19, β = −0.46, (t = −9.12, *p* < 0.001)). This showed a negative relation between both variables.

## 4. Discussion

Taking everything into account, the reduced version of the CAMM was proved to have good reliability and appears suitable to be used in Spanish-native samples. Results suggest that Spanish 5-item CAMM is a developmentally appropriate DM measure with adequate internal consistency.

Consistent with our findings, several studies found evidences of the benefits of a reduced version of CAMM [8,24,31,32,33,34,35]. Our conclusions, in common with those of the other similar investigations, suggest that a new version of CAMM may be used as a simple and flexible instrument to measure dispositional mindfulness in mindfulness-based training for children and adolescents [35,48]. 

First, we discuss reliability of the 10-iem CAMM. Since there are items with negative or very low correlation with the total score of 10-item CAMM, it seems that individuals are misinterpreting those items. This view is sustained with the ANOVAs results showing differences in total scores as age increases. This could be interpreted as whilst children grow, their verbal reasoning and comprehension of the meaning of the items improves [49]. The writing of the items expressed in a negative way may hinder the understanding of children, however, adolescents are more qualified to do so. Especially, children have more difficulties to understand negative sentences than positive ones [50,51]. These are the final 5-item scale, and 2,5 and 10 are the first items to be removed. 

Regarding the analysis of components, despite having tested a large number of models, none of them satisfied the criteria for their viability, except for the one-factor finding with the CAMM reduced to five items. This model has a superb fit to the data according to CFA experts [44]. In addition, different samples have been used for the EFA and the CFA, in order to avoid potential validity problems. Overall, regarding other studies, a general tendency exists to suggest excluding some items—especially #2, 5, and 10 [31,32,33,34]. Meanwhile, in this study the best CAMM factor structure for this brief Spanish version was constituted by five items (# 1,4,7,8,9 see Appendix A). 

## 5. Conclusions

As expected, a reduced version of the CAMM showed better predictive validity with negative affect and thought suppression. Thus, in this study, individuals with higher levels of DM tend to have lower tendencies to suppress thoughts, in line with the previous studies [1,4,8,15,52,53,54]. Although in our study, DM did not tend to perceive a higher quality of feeling positive affect, DM was negatively related to negative affect. DM and negative affect are consistently related each other according to several studies [4,55,56,57,58].

Reliability and construct validity are granted in the final 5-item reduced scale. Reliability also improves in spite of the fact we cut the test by half, which gives an idea of how little the deleted items contribute to the whole scale. The factor underlying the original model of the CAMM should be considered as an indisposition to mindfulness, since the wording in negation of the items seems to point in this direction. Therefore, the CAMM’s items work in an inverse manner, which makes their comprehension considerably more difficult, especially at younger ages, because of cognitive development [51]. 

In spite of these findings, our research presents a limitation that needs to be underlined with respect to the generalization of the results. The study was carried out with a sample of Spanish subjects. Future research extending this sample would help the generalization, so it would be necessary to replicate this study in a larger population to further analyze this variable.

As regards to future studies, it is recommended to develop a new version of the Spanish CAMM version for children and adolescents, whose items are written directly instead of negative statements. To check whether their psychometric properties improve and convergent validity with other measuring instruments, for instance, with positive psychology topics. This will imply a better understanding and facilitate the psychological processes that lead to support an element, given a relevant part of cognitive psychology during childhood and adolescence. It would also be interesting to compare the shorten Spanish CAMM with this last suggested Spanish CAMM version, and test whether writing items in direct dispositional instead of in indispositional mindfulness would affect to relationships with other related topics and criteria.

## Figures and Tables

**Table 1:** Total Variance Explained-Exploratory Factor Analysis (EFA) for 7-items CAMM (using Valencia sample, *n*_1_ = 360).

Component	Initial Eigenvalues			Rotation Sums of Squared Loadings
	Total	% of Variance	Cumulative %	Total
1	3.27	46.65	46.66	3.10
2	1.21	17.28	63.94	1.97

**Table 2:** Descriptive Statistics for every single version of Spanish CAMM (*n* = 678). Predict validity study (correlations) was conducted using Andalusian sample (*n* = 318). Cronbach’s alpha reliability is reported (alpha). No significant differences between male and female participants for all measures were found. In bold significative relationships.

	Min/Max	M 8 (*SD*)	α	1	2	3	4	5	6	7
1 Age	8/16	12.01 (1.730)		*						
2 10-item CAMM	4.00/40.00	26.09 (5.55)	0.67	0.14 **	*					
3 7-item CAMM	0.00/28.00	19.41 (4.78)	0.77	0.17 **	0.92 **	*				
4 5-item CAMM	0.00/20.00	14.45 (3.78)	0.76	0.25 **	0.84 **	0.94 **	*			
5 PA PANASN	10.00/30.00	23.48 (3.74)	0.72	−0.05	−0.04	0.02	0.02	*		
6 NA PANASN	10.00/26.00	16.12 (3.89)	0.78	−0.11 *	−0.47 **	−0.48 **	−0.46 **	−0.15 **	*	
7 Bal. PANASN	−8.00/20.00	7.36 (5.78)	0.65	0.04	−0.29 **	0.34 **	0.32 **	0.75 **	−0.77 **	*
8 TS WBSI	15.00/74.00	46.62 (13.21)	0.88	−0.14 *	−0.58 **	−0.59 **	−0.61 **	0.04	0.35 **	−0.21 **

* *p* < 0.05; ** *p* < 0.01; PA: positive affect; NA: negative affect; Bal.: Balance (PA-NA) of PANASN. TS: Thought suppression.

## Appendix A. Child and Adolescent Mindfulness Measure (CAMM), Original and SPANISH Version (in Italics)

**Table:**

CAMM 1 *	I get upset with myself for having feelings that don’t make sense
	*Me siento mal conmigo mismo por tener sentimientos que no tienen sentido*
CAMM 2	At school, I walk from class to class without noticing what I’m doing
	*En la escuela, camino de clase a clase sin darme cuenta de lo que estoy haciendo*
CAMM 3	I keep myself busy so I don’t notice my thoughts or feelings
	*Me mantengo ocupado por lo que no soy consciente de mis pensamientos o sentimientos*
CAMM 4 *	I tell myself that I shouldn’t feel the way I’m feeling
	*Me digo a mí mismo que no debería sentir lo que estoy sintiendo*
CAMM 5	I push away thoughts that I don’t like
	*Aparto de mi mente los pensamientos que no me gustan*
CAMM 6	It’s hard for me to pay attention to only one thing at a time
	*Es difícil para mí prestar atención a una sola cosa en un momento dado*
CAMM 7 *	I get upset with myself for having certain thoughts
	*Me molesto conmigo mismo por tener ciertos pensamientos*
CAMM 8 *	I think about things that have happened in the past instead of thinking about things that are happening right now
	*Pienso sobre cosas que han pasado en el pasado en vez de pensar en cosas que están pasando ahora mismo*
CAMM 9 *	I think that some of my feelings are bad and that I shouldn’t have them
	*Pienso que algunos de mis sentimientos son malos y no debería tenerlos*
CAMM 10	I stop myself from having feelings that I don’t like
	*Me abstengo de tener sentimientos que no me gustan*

* Results showed that the best CAMM factor structure was constituted by these five items.
